# Geographical Distribution and New Situation of* Leishmania* Species after the Control of Cutaneous Leishmaniasis Foci in Errachidia Province, Morocco, in 2014

**DOI:** 10.1155/2016/8642373

**Published:** 2016-06-28

**Authors:** El Alem Mohamed Mahmoud, Sebti Faiza, Med Lemine, Chichaoui Smaine, El Bachir Adlaoui, Habbari Khalid, Sadak Abderrahim, Fellah Hajiba

**Affiliations:** ^1^National Reference Laboratory of Leishmaniasis, National Institute of Hygiene, 27 Avenue Ibn Battuta, Agdal, 11400 Rabat, Morocco; ^2^Laboratory of Zoology and General Biology, Faculty of Sciences, Mohammed V University in Rabat, Avenue Ibn Battouta, BP 1014, 10000 Rabat, Morocco; ^3^Faculty of Sciences, Mohammed V University in Rabat, Avenue Ibn Battouta, BP 1014, 10000 Rabat, Morocco; ^4^Ministry of Health, Bd-Bir-Anzarane, BP 57, 52000 Errachidia, Morocco; ^5^Department of Parasitology, National Institute of Hygiene, 27 Avenue Ibn Battuta, Agdal, 11400 Rabat, Morocco; ^6^Faculty of Sciences and Technics, University Sultan Moulay Slimane, Boulevard Ibn Khaldoun, 23000 Béni Mellal, Morocco

## Abstract

In Errachidia province, the incidence of cutaneous leishmaniasis (CL) has increased over the past decade and it was higher in 2010 (860.34 per 100,000 inhabitants), with 3445 cases. The number of cases declined sharply and decreased from 3445 cases in 2010 to 8 cases in 2014 following the control action plan interventions. The total of patients was diagnosed only on clinical basis and the lesions were considered caused by* L. major*. The epidemiological study was conducted between 2001 and 2014 and the molecular detection of CL was studied to identify the circulating parasite species in this province by using the ITS1-PCR-RFLP methods. In 2010, the molecular identification of 11 samples revealed the presence of* L. major* in the most affected circles: Goulmima, Er-Rissani, and Errachidia. In 2014 the molecular characterization of 7 among 8 cases reported in this year showed the presence of* L. tropica* in Errachidia circle. This is the first molecular identification of* L. tropica* in Errachidia province. The detection of this species after the intensified control measures strategies suggests that it was probably dissipated through the dominance of* L. major*.

## 1. Introduction

**Figure 1:**
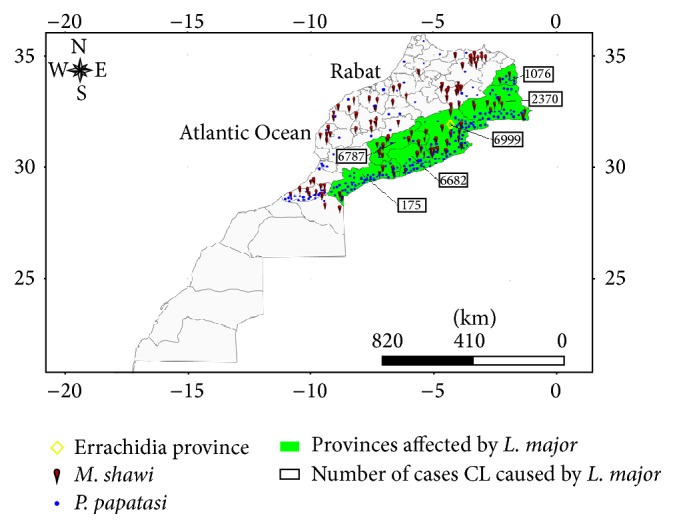
Repartition of* M. shawi* rodent and densities of* P. papatasi* and CL caused by* L. major *distributions in Morocco from 2001 to 2014 [1, 7, 10].

In Morocco, cutaneous leishmaniasis (CL) is caused by three species of* Leishmania*:* L. major*,* L. tropica,* and* L. infantum.* CL due to* L. major* is considered as a major public health threat. The clinical characteristic is a single localized cutaneous lesion, which is often severely inflamed as well as ulcerated and healing within 2–8 months, but a polymorphism can be observed [1, 2]. This disease is characterized by its wide geographical distribution in arid zones in the palm groves of the southern foothills of the Anti-Atlas and High Atlas, especially in Jerada, Figuig, Errachidia, Ouarzazate, Zagora, and Tata provinces (Figure 1) [1]. The distribution of* L. major *is conditioned by the presence of suitable reservoir hosts mainly the commensal rodent* Meriones shawi *(*M. shawi*) and sand fly vector mainly* Phlebotomus papatasi* [3]. The parasite-reservoir host combination was subsequently observed by formal identification of the parasite [4–6]. This reservoir (*M. shawi* rodent) is the most ubiquitous species in Morocco and its distribution largely exceeds* L. major* repartition [7], proving its role in transmission of disease. In terms of vectors, the bioclimate affects the vectors distribution and density and hence disease prevalence [8];* P. papatasi* is the main vector of* L. major,* found common to all environments but with a varied predominance, especially in saharan environment where it was the most prevalent species [9]. The CL caused by* L. major* in Morocco is located in the east of the country [1], which is consistent also with the high densities of vectors and rodents in the south and southeast of country according to Laqraa et al. [10] and Echchakery et al. [7], respectively. The* M. shawi-P*.* papatasi*-*L. major* association seems to constitute quite stable zoonotic systems as shown in Figure 1. Isoenzymatic characterization of* L. major* indicated the presence of a unique zymodeme MON-25 [8]. In 2010, the number of* L. major* cases saw a great increase with 6444 cases; the highest rate was recorded in Errachidia province including over than 3000 cases recorded. In 2011, the number of cases saw a large decrease following the control action plan interventions. In the present study, after the new epidemiological situation and a better control of leishmaniasis adopted, an epidemiological study and ITS1-PCR method were used for update and identification of* Leishmania* parasite species responsible for the recent CL cases in Errachidia province.

## 2. Materials and Methods

### 2.1. Study Area

Errachidia is a province located in the south east of Morocco (31°45′0′′N/4°30′0′′W), which was separated administratively since 2009. This province is constituted by four circles currently having 29 communes with 7 urban and 22 rural communes. This province covers an area of 59,585 km^2^; it is bordered in the north by Midelt province, in the northeast by Figuig province, in the south and southeast by Algeria, and in the west by Tinghir and Zagoura provinces. The climate is semidesert; maximum and minimum mean temperatures in Errachidia amount to 5°C in January and 31.5°C in July. With low rainfall and spread of irregular way in time and space, most of the territory sits down within 100 mm of rain per year. Due to its typical climate, it is characterized by low density of vegetation cover. The total population amounted to 418,451 in 2014 [11]. The rural population makes out around 53.61% with an overall density of 7.02 inhabitants per square kilometer [11].

### 2.2. Sampling and Diagnosis

Samples of DNA were isolated from the positive skin smears in 2010, for 11 CL lesions performed in Errachidia, Goulmima, and Er-Rissani circles which are the most affected. In 2014 we collected 7 skin smears of CL from 8 cases only recorded in circle of Errachidia. These slides were examined by the provincial laboratory and sent to the national laboratory of leishmaniasis in National Institute of Hygiene for control and confirmation. Smears (Giemsa-stained slides) were prepared from skin lesions and examined microscopically for the detection of* Leishmania* amastigotes.

### 2.3. Extraction of DNA from Stained Smears and PCR Analysis

DNA was extracted and purified from positive smears using a kit “high pure template PCR” according to the instructions of the manufacturer. The ribosomal internal transcribed spacer (ITS1) region was analyzed using the primer pair L5.8S and LITSR by PCR-restriction fragment length polymorphism (RFLP) approach for identification of the* Leishmania* parasites. The product was loaded and analyzed on 1.5% agarose gels by electrophoresis and visualized by UV lights [13]. Positive controls contain DNA of* L. infantum* (MHOM/MA/1998/LVTA),* L. tropica* (MHOM/MA/2010/LCTIOK-4), and* L. major* (MHOM/MA/2009/LCER19-09). Negative controls (distilled water) were included during PCR to ensure reliability and validity and to check for possible contaminations of the amplification reactions. PCR product was followed with the RFLP analysis using Hae III enzyme.

### 2.4. Data Analysis

Epidemiological data were exploited by using Microsoft Office Excel 2010 and analyzed by R Software version 3.2.2. We used chi-square tests to compare proportions. Quantum Geographic Information System (GIS) was used to design and develop CL distribution maps.

## 3. Results

**Figure 2:**
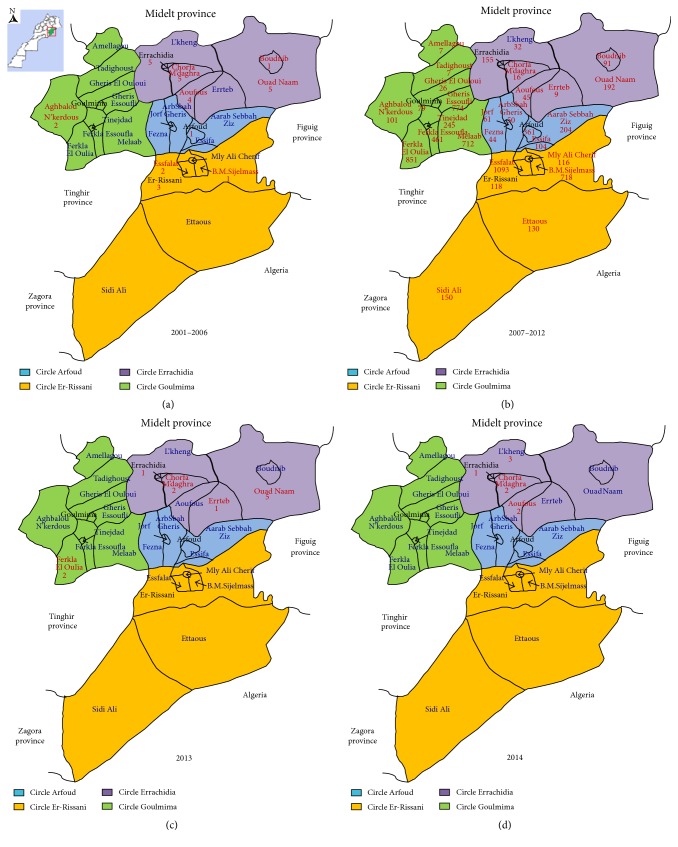
Map of Morocco showing Errachidia province. The study area and geographical distribution of CL from 2001 to 2014. The four circles of Errachidia province following the administrative map are colored by green, purple, blue, and orange.

**Figure 3:**
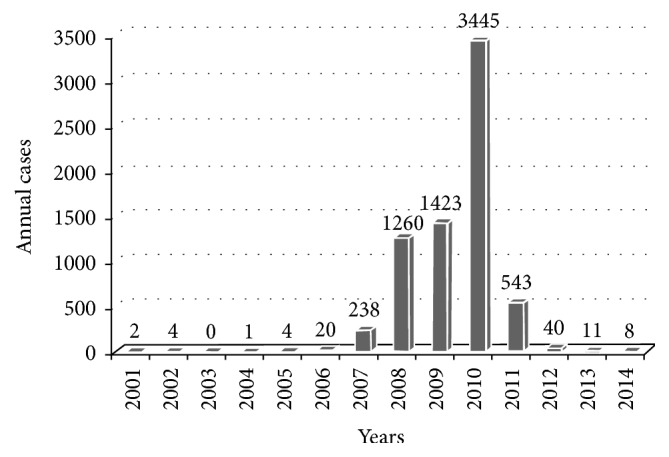
Evolution of CL cases in Errachidia province showing cumulative number per year for the period 2001 to 2014, with major peak in 2010 [12].

**Figure 4:**
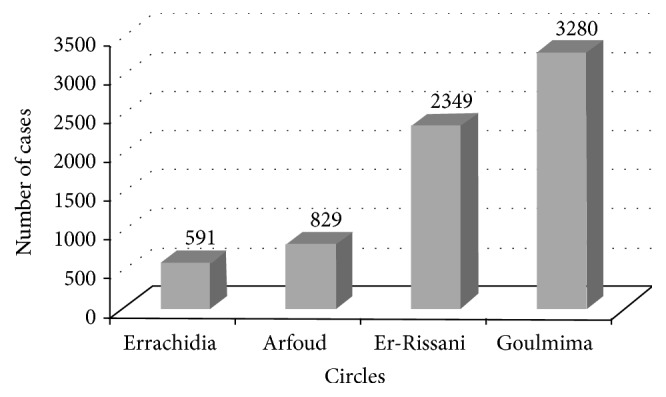
Evolution of CL cases in the four circles of Errachidia province from 2001 to 2014.

During the period 2001–2014, there were a total of 7653 CL cases notified in Errachidia province. The epidemiologic situation remained stable until the year 2004 with an average of 2 cases per year; over 2007–2014 the number of cases had an exponential increase with fluctuations. Period from 2007 to 2011 has known a great epidemic. The number of cases has decreased slightly in the following years, with a new recrudescence until 8 cases in 2014. The maximum incidence peak was reached in 2010 with 860.34/100,000 inhabitants (3445 cases) (Figure 3). The spatial as well as temporal distribution of CL cases in the four circles of Errachidia province is illustrated in Figure 2. The first recorded CL cases appeared in 2001 towards the end of 2006 (Figure 2(a)). In the following years (2007–2012), the outbreak spread to the north and west of the province towards Sahara in the south and extending to the Algerian border. We observed higher rates of CL (over 1000 cases) in the four circles, while the highest rate for CL was found in northern of the province (Figure 2(b)). Considering its recent control, CL cases dropped to 11 cases for the rest of 2013 (Figure 2(c)). CL case numbers subsequently decreased during 2014; the few cases recorded are located in the north of the province at the Errachidia circle (Figure 2(d)). Among four circles in this province, the temporal and spatial distribution of CL recorded from 2001 to 2014 showed that 79.86% of cases were recorded in Goulmima and Er-Rissani circles (3 urban and 13 rural districts among 29 total districts) and 11.76% and 8.38% were declared, respectively, in Arfoud and Errachidia circles (Figure 4).

### 3.1. Distribution of CL by Age and Sex

**Figure 5:**
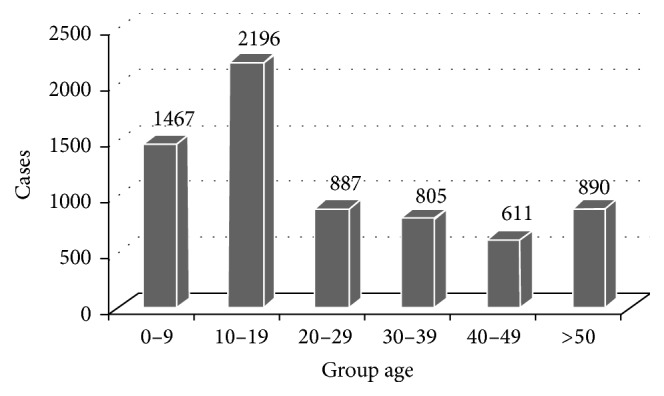
Distribution of CL according to age from 2001 to 2014 in Errachidia province.

Viewing the important CL cases recorded in Errachidia, we were able to make an exploitation of the patient files and represent the distribution of cases by age and sex. Usually, all ages are affected by the disease, with a high prevalence for age of 10–19 years (32.03%) followed by the age of 0–9 years (21.39%). Other age groups (from 20–39 to over 50 years) were weakly affected with, respectively, 12.93%, 11.74%, and 12.98%. However, age group of 40–49% is less affected with 8.91% (Figure 5).

The difference of CL cases from 10 to 19 year old was statistically significant in regard to the other age groups (*χ* ^2^ = 1523.3, *p* value < 2.2*e* − 16).

Both sexes are affected by CL, and females were more affected with a sex ratio of F/M = 1.44. The difference between males and females was statistically significant (*χ* ^2^ = 229.55, *p* value < 2.2*e* − 16).

### 3.2. Results of PCR

The results of the molecular study of 11 samples collected in 2010 showed that CL was due to* L. major* in Errachidia, Er-Rissani, and Goulmima circles. In 2014, the molecular identification of 7 among the 8 cases reported in the Errachidia circles showed the presence of only* L. tropica *(Table 1, Figures 2 and 7).

## 4. Discussion

Since the appearance of CL in 1914 in Morocco, the numbers of cases have showed some fluctuation. In recent years the diagnosis of patients was performed only on clinical basis; at the national level, the highest numbers of CL cases due to* L. major* were noted in 2010 (6444 cases), among them nearly 50% (3445 cases) were recorded in Errachidia province [12]. The Ministry of Health has decided to implement the response strategy in the fight against leishmaniasis based mainly on diagnosis and treatment of patients, vector, and reservoir control measures for reducing the incidence of disease. Besides the literature, recent study shows that the best way to control* L. major* is to combine reservoir and vector control [14]. In Errachidia, the rodents control was targeted for control policy of CL; the control of vectors and reservoir hosts is based on improving hygiene conditions and chemical measures for controlling rodent populations by using the rodenticides giving beneficial results in the active burrows in all infested areas. However, the systematic application of poisoned bait in the CL-affected areas in Errachidia province has probably contributed to the decrease in CL incidence, as it led to a 95% reduction in the number of active rodent burrows in treated areas, which was sustained for each of the 3 years of the intervention [15]. In addition, all these measures of controlling actions are supported by education and raising public awareness with collaboration of local collectives and associations.

The data show that Goulmima and Er-Rissani circles were the most affected by CL. This could possibly be explained by the dominance of rural area of high densities of populations near to* L. major* reservoir host biotopes. These areas host the largest oases of Errachidia, namely, Ferkla, Tinjdad, Goulmima, and Er-Rissani oasis, which are adjacent to the great watersheds Ziz and Rhéris of Errachidia. Therefore in arid areas, the huge mobilization of water resources and the creation of new irrigated zones probably favored the proliferation of both sandflies and rodents, including gerbils and jirds living in burrows with* P. papatasi*, the vector of* L. major *[16]. This rodent (the main reservoir host of* L. major*) is considered as an agricultural pest.* M. shawi *is the main infected animal in south Morocco [17]. Most recent studies show in the rural area poorer farmers might live close to their animals, which promotes humidity and the synthesis of organic material for breeding of sandfly [2].

During this study designed to evaluate the overall situation of CL and molecular identification of circulating species in the Errachidia province, we noted clearly that the number of cases was significantly declined during the last 4 years; this decline is due to the outbreak control measures focusing on rodents and sandflies control. According to age, the majority of the cases occurred in an age group less than 19 years; this could be explained by their weak immunity system. In general, CL tends to affect a younger age group because they are consistently more exposed to phlebotomine sandfly bite by their habit to play near breeding sites [18]. However, the majority of cases infected were diagnosed by passive detection, which could possibly explain the higher rate of infection in women (sex ratio F/M = 1.44) over men, by the fact that women consult more than men because of the unsightly lesions [19, 20].

Frequently in all provinces known by CL due to* L. major*, the* Leishmania* species are identified based on their geographical distribution and on the clinical manifestations. The diagnosis of the infecting species based on clinical symptoms is not crucial, because symptoms can vary and may be confused with other etiologic agents [21]. In Morocco and particularly in Errachidia province, the diagnosis is done only by the clinical methods. The “wet” type of* L. major* is a classical lesion aspect, associated with infiltrated nodule causing the localized cutaneous lesions (LCL) form. These various clinical manifestations of* L. major* are dug which can evolve into an ulceration covered by crust. It is also often severely inflamed, ulcerated, and healing within two to eight months. In addition, a remarkable polymorphism of lesions caused by* L. major* was also observed with 11 different forms (vegetative, impetiginoid, eryseloid, necrotic, warty, erythematosquamous, lupoid, sporotrichoid, papulous, eczematoid and recidivans) [1, 18, 22–27].

Conversely, the lesions caused by* L. tropica* are dry, lupoid, small (2 cm in diameter), unique, self-healing, and mainly located on the face and can last up to a year [28, 29]. A clinical polymorphism of this cutaneous form was also described with eight different forms (impetiginized, ulcerocrusted, noduloulcerative, severe, vegetant inflammatory, large, multiple and limbs infections) [1, 30]. According to these data concerning this clinical manifestation the confirmation of the circulation of* Leishmania* species cannot be differentiated and detected without molecular biology analysis.

**Figure 6:**
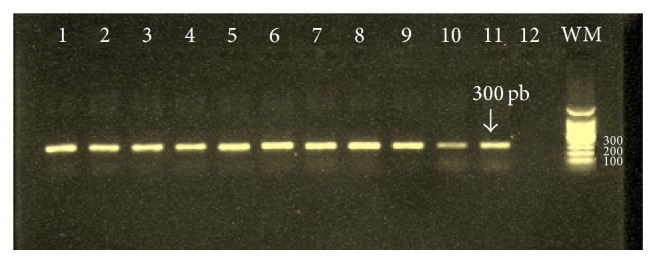
Gel electrophoresis of* Leishmania *(ITS1-PCR product) from Giemsa-stained lesion smears isolates obtained from patients. Lanes 1–10 correspond to clinical materials from patients. Sample from slides, lane 11: positive control, lane 12: negative control, and WM: weight marker, 100 pb.

**Figure 7:**
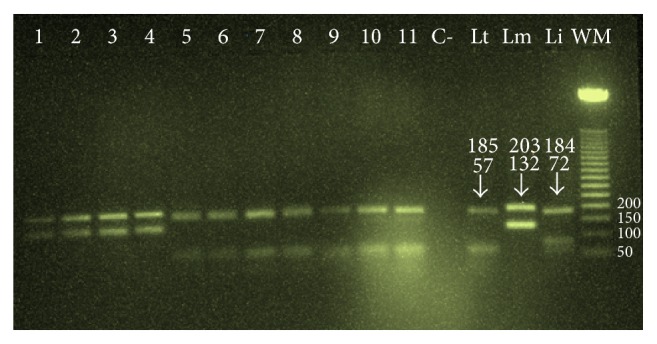
Agarose gel electrophoresis analysis of ITS1-PCR-RFLP amplified products from different slides collected, lanes 1–4 (*L. major* detected in the samples 2010), and lanes 5–11 (*L. tropica* detected in the samples 2014), C: negative control, Lt:* L. tropica,* Lm:* L. major,* Li:* L. infantum, *and WM: weight marker (50 pb).

On the other hand, molecular identification revealed for the first time the presence of* L. tropica *(Figures 6 and 7). We think that the presence of this species could be related to semiarid climate in part of High Atlas in this region where the conditions are favorable, which is consistent with the weak distribution of its vector* P. sergenti *[10]. Therefore, we can say that* L. tropica* was present but at low density compared to* L. major*.

Our study has some limitations. Case is based on physicians' expertise and the availability of laboratory facilities with trained staff. Unfortunately, it was not possible to conduct the most of skin lesion smears during the epidemic period especially in 2010.

## 5. Conclusion 

These results show the success of the implementation of the response strategy which reduced dramatically* L. major* CL. However, molecular analysis of persistent cases showed the existence of* L. tropica* which was probably dissipated through the dominance of* L. major*.

## Figures and Tables

**Table 1:** Results of molecular study of slides of the most affected circle in Errachidia province.

Province	Year	Circle	Commune	Results of ITS1-PCR	Results of RFLP by HaeIII
*Errachidia*	2014		L'kheng	Positive	*L. tropica*
	2014		L'kheng	Positive	*L. tropica*
	2014		M'daghra	Positive	*L. tropica*
	2014	*Errachidia*	Aoufous	Positive	*L. tropica*
	2014		Errachidia	Positive	*L. tropica*
	2014		Errachidia	Positive	*L. tropica*
	2014		M'daghra	Positive	*L. tropica*

*Errachidia*	2010		Er-Rissani	Positive	*L. major*
	2010		Er-Rissani	Positive	*L. major*
	2010	*Er-Rissani*	Er-Rissani	Positive	*L. major*
	2010		Er-Rissani	Positive	*L. major*
	2010		Er-Rissani	Positive	*L. major*

*Errachidia*	2010		Goulmima	Positive	*L. major*
	2010	*Goulmima*	Goulmima	Positive	*L. major*
	2010		Goulmima	Positive	*L. major*

*Errachidia*	2010		Errachidia	Positive	*L. major*
	2010	*Errachidia*	Errachidia	Positive	*L. major*
	2010		Errachidia	Positive	*L. major*

## References

[1] Rhajaoui M., Nasereddin A., Fellah H. (2007). New clinicoepidemiologic profile of cutaneous leishmaniasis, Morocco. *Emerging Infectious Diseases*.

[2] World Health Organization (2010). *Control of the Leishmaniasis: Report of a Meeting of the WHO Expert Committee on the Control of Leishmaniases, Geneva, 22–26 March 2010*.

[3] Prudhomme J., Gunay F., Rahola N. (2012). Wing size and shape variation of *Phlebotomus papatasi* (Diptera: Psychodidae) populations from the south and north slopes of the Atlas Mountains in Morocco. *Journal of Vector Ecology*.

[4] Ashford R. W., Schnur L. F., Chance M. L., Samaan S. A., Ahmed H. N. (1977). Cutaneous leishmaniasis in the Libyan Arab Republic: preliminary ecological findings. *Annals of Tropical Medicine and Parasitology*.

[5] Belazzoug S. (1983). Isolation of Leishmania major Yakimoff & Schokhor, 1914 from *Psammomys obesus* Gretzschmar, 1828 (Rodentia: Gerbillidae) in Algeria. *Transactions of the Royal Society of Tropical Medicine and Hygiene*.

[6] Ben Ismail R., Ben Rachid M. S., Gradoni L., Gramiccia M., Helal H., Bach-Hamba D. (1987). La Leishmaniose cutanée zoonotique en Tunisie Etude du réservoir dansle foyer de Douara. *Annales de la Societe Belge de Medecine Tropicale*.

[7] Echchakery M., Boussaa S., Kahime K., Boumezzough A. (2015). Epidemiological role of a rodent in Morocco: case of cutaneous leishmaniasis. *Asian Pacific Journal of Tropical Disease*.

[8] Rioux J. A., Rispail P., Lanotte G., Lepart J. (1984). Relations Phlébotomes-bioclimats en écologie des leishmanioses Corollaires épidémiologiques. L'exemple du Maroc. *Bulletin de la Société Botanique de France*.

[9] Faraj C., Ouahabi S., Adlaoui E. B. (2012). Insecticide susceptibility status of *Phlebotomus* (*Paraphlebotomus*) *sergenti* and *Phlebotomus* (*Phlebotomus*) *papatasi* in endemic foci of cutaneous leishmaniasis in Morocco. *Parasites and Vectors*.

[10] Laqraa E., Elkohli M., Adlaoui E., Faraj C. (2015). *Les Phlébotomes du Maroc: Bulletin de L'institut National D'hygiene*.

[12] Moroccan Ministry of Health (2012). *Etat Davancement des Programmes de Luttecontre les Maladies Parasitaires*.

[13] Schönian G., Nasereddin A., Dinse N. (2003). PCR diagnosis and characterization of Leishmania in local and imported clinical samples. *Diagnostic Microbiology and Infectious Disease*.

[14] Reithinger R., Dujardin J.-C., Louzir H., Pirmez C., Alexander B., Brooker S. (2007). Cutaneous leishmaniasis. *The Lancet Infectious Diseases*.

[15] Bennis I., De Brouwere V., Ameur B. (2015). Control of cutaneous leishmaniasis caused by *Leishmania major* in south-eastern Morocco. *Tropical Medicine and International Health*.

[16] Aoun K., Tiouiri H., Ghrab J., Boufaroua M. (2004). Lacs collinaires et santé humaine: quelle situation en Tunisie?. *Microbiologie et Hygiène Alimentaire*.

[17] Rioux J. A., Petter F., Akalay O. (1982). Meriones shawi (Duvernoy, 1842) [Rodentia, Gerbillidae] réservoir de Leishmania major, Yakimoff and Schokhor, 1914 [Kinetoplastida, Trypanosomatidae] au Sud Maroc. *Comptes Rendus de l'Académie des Sciences, Serie III: Sciences de la Vie*.

[18] Zait H., Bousaad H. (2009). Leishmanioses cutanées en Algérie Bilan de 386 cas diagnostiqués au CHU Mustapha d'Alger de 1998 à 2007. *Revue Francophone Laboratoires*.

[19] Arroub H., Alaoui A., Lemrani M., Habbari K. (2012). Cutaneous leishmaniasis in foum jamâa (Azilal, Morocco): micro-environmental and socio-economical risk factors. *Journal of Agriculture & Social Sciences*.

[20] Chiheb S., Guessous-Idrissi N., Hamdani A. (1999). Clinical features of cutaneous leishmaniasis due to Leishmania tropica in an emerging focus in North Morocco. *Annales de Dermatologie et de Venereologie*.

[21] Rhajaoui M. (2011). Human leishmaniases in Morocco: a nosogeographical diversity. *Pathologie Biologie*.

[22] Abdellatif M. Z. M., El-Mabrouk K., Ewis A. A. (2013). An epidemiological study of cutaneous leishmaniasis in Al-jabal Al-gharbi libya. *Korean Journal of Parasitology*.

[23] Adler S., Ber M. (1941). The transmission of Leishmania tropica by the bite of P. papatasi. *Indian Journal of Medical Research*.

[24] El-Buni A. A., Edwebi H., Ben Darif A. L. (1997). Prospective study among cutaneous leishmaniasis cases in Tripoli Central Hospital, Tripoli, Libya. *Archives de l'Institut Pasteur de Tunis*.

[25] Masmoudi A., Kidar A., Rebai M., Bouassida S., Turki H., Zahaf A. (2005). La Leishmaniose cutanée de la face dans la région de Gafsa, Tunisie. *Distribution Manuscrit No.*.

[26] Rioux J. A., Lanotte G., Petter F., Rioux J. A. (1986). Les leishmanioses cutanées du bassin Méditerranéen occidental. De l'identification enzymatique à l'analyse éco-épidémiologique. L'exemple de trois ‘foyers’, tunisien, marocain et français. *Leishmania taxonomie et phylogénèse. Applications éco-épidémiologiques. Colloque International Centre National de la Recherche Scientifique, Institut National de la Santé et de la Recherche Médicale (CNRS INSERM) 1984*.

[27] Ysmail-Dahlouk M., Ammar Khodja A., Ysmail-Dahlouk S., Ait Belkacem F. (1994). Leishmaniose lupoide. *Annales de Dermatologie et de Vénéréologie*.

[28] Chiheb S., Hamdani A., Riyad M., Bichichi M., Hamdani S., Krimech A. (1999). Cuta-neous leishmaniasis due to Leishmania tropica; Clinical features in a new focusin northern Morocco. *Annales de Dermatologie et de Vénéréologie*.

[29] Marty P., Le Fichoux Y., Pratlong F., Rioux J. A., Rostain G., Lacour J. P. (1989). Cutaneous leishmaniasis due to *Leishmania tropica* in a young Moroccan child observed in Nice, France. *Transactions of the Royal Society of Tropical Medicine and Hygiene*.

[30] Guessous-Idrissi N., Chiheb S., Hamdani A. (1997). Cutaneous leishmaniasis: an emerging epidemic focus of *Leishmania tropica* in north Morocco. *Transactions of the Royal Society of Tropical Medicine and Hygiene*.

